# Changes in Morphological, Physiological and Phytochemical Traits of Different Dill (*Anethum graveolens* L.) Cultivars as Affected by Light-Emitting Diodes

**DOI:** 10.3390/molecules29235506

**Published:** 2024-11-21

**Authors:** Nafiseh Dehghani, Maryam Haghighi, Mehdi Rahimmalek, Mohammad R. Sabzalian, Antoni Szumny

**Affiliations:** 1 Department of Horticulture, College of Agriculture, Isfahan University of Technology, Isfahan 84156-83111, Iran; dehghaninafi3.78@gmail.com (N.D.); mhaghighi@cc.iut.ac.ir (M.H.); 2 Department of Food Chemistry and Biocatalysis, Wrocław University of Environmental and Life Sciences, 50-375 Wrocław, Poland; 3 Department of Agronomy and Plant Breeding, College of Agriculture, Isfahan University of Technology, Isfahan 84156-83111, Iran; sabzalian@iut.ac.ir

**Keywords:** essential compounds, medicinal plants, secondary metabolites, supplementary light

## Abstract

Dill is a fragrant vegetable containing various beneficial compounds for health. This research aims to evaluate the impact of various spectra of LED light on essential oil composition and morphological and physiological characteristics of three dill cultivars. LED light treatments included greenhouse light as control (C), blue (B), red (R), red + blue (RB), and white (W). RB light enhanced most physiological indicators investigated in this study, including photosynthetic pigments, phenols, flavonoids, and antioxidant capacity. Furthermore, electrolyte leakage in the three cultivars of Khomein, Isfahan, and Varamin decreased when exposed to RB light compared with C light. Under RB light, the essential oil contained more dill ether and α-phellandrene than in other light conditions. In general, light treatment with 75% R light and 25% B light had a noticeable impact on enhancing physiological features compared with other light spectrums. α-phellandrene levels increased in the Isfahan and Varamin cultivars under RB and B light conditions. Finally, the RB light and Khomein cultivars improved physiological features, whereas RB and R light in the Varamin and Isfahan cultivars are recommended for more essential oil compositions in functional food production.

## 1. Introduction

Recently, interest in aromatic and therapeutic plants has grown among manufacturers and consumers due to their fragrant, medicinal, and preservation properties [1]. These plants are utilized in various applications, including nutraceuticals, herbal medicine, aromatherapy, and other therapeutic practices [2]. Dill (*Anethum graveolens* L.) is a leafy plant that originates from southern Europe and West Asia and is commonly found growing naturally in the Mediterranean region [3]. Dill is not only used as a culinary herb to enhance flavors, but it also possesses a range of medicinal properties. The medicinal qualities of dill include antioxidant, antibacterial, antifungal, anti-inflammatory, and organprotective effects [4]. Dill, both dried and fresh, is used to treat some diseases because of its helpful essential oils, which are most concentrated when the plant is harvested at the flowering stage [5]. Researchers have recently become interested in the impacts of essential oils on various systems in the body, such as the respiratory, gastrointestinal, neurological, and immune systems, along with their abilities to fight off microbes and fungi [6,7]. Secondary metabolites produced by plants, including essential oils derived from plants that are safe to eat and historically used plants, are typically considered safer compared to artificial compounds as they are mostly biodegradable and have not been associated with any adverse effects. Therefore, the discovery of innovative and secure antibacterial and anticancer medications sourced from nature is becoming increasingly important. Vegetables are preferred for consumption because of their nutritional content. In plant factories, methods are employed to increase certain secondary metabolites, such as essential oils from vegetables like dill, to increase nutraceutical values [7,8]. The dill plant has different cultivars; however, in Iran, only the legume variety is registered on the website of the Institute of Registration and Certification of Seeds and Seedlings.

Depending on the parts of the plant and their growth phase, the composition of dill’s basic oils is different. The main components are α-phellandrene, limonene, β-phellandrene, and p-cymene in the vegetative phase. However, p-cymene, carvone, and dill ether were identified as the primary components during the flowering period [9].

It is well-known that climatic conditions have a significant impact not only on the growth and shape of plants but also on the production of medicinal essential oils and fragrant plants [10]. Although the content of essential oils is predominantly determined by genetics [11], their creation is also heavily affected by environmental factors like the length of the day irradiance, temperature, water availability, and light [12,13]. They control the buildup of plant compounds, for example, flavonoids, ascorbic acid, carotenoids, tocopherol, enzyme antioxidants, and the production of essential oils [14].

Light is an essential non-living element that greatly impacts the rate of growth and advancement of plants. It powers the photosynthesis process and also controls various other processes [4]. The plant’s physiology is influenced by three main characteristics of light: the amount (intensity and duration), quality (spectral composition), and distribution. Recent studies have highlighted the significant role of light’s spectral composition in driving specialized plant metabolism [4]. Horticultural LED modules have become increasingly popular as artificial grow lights, serving either as supplementary light in the greenhouse or as the primary light source in indoor plant factory setups that maintain controlled environmental conditions [15].

LEDs can enhance the production of oils and secondary compounds in plants [16,17]. According to reports, the range of light wavelengths coordinately alters the makeup of these chemicals in essential oils. This suggests exposure to specific light spectra may change the substances found in bioactive molecules [18]. For example, B light produced the largest quantity of basil leaf essential oil [19]. *Thymus migricus* had the highest thymol concentration if illuminated by B LED light [17]. Sabzalian et al. [20] found that *Mentha longifolia* essential oil production increased fourfold upon being subjected to RB LED light. Johkan et al. [21] similarly found that lettuce seedlings exposed to B LED light had stronger antioxidant activity than those treated with R or fluorescent lighting. Differences in plant cultivars contribute to variations in both the amount and the characteristics of secondary metabolites, leading to notable differences in their production levels [22]. Research indicates that essential oil composition production and output may fluctuate depending on the plant cultivar [23,24].

This study assesses the effect of various LED light spectra on secondary metabolic composition and changes in morphology, chemical composition, and physiological characteristics of three cultivars of dill: Khomin, Isfahan, and Varamin.

## 2. Results and Discussion

### 2.1. Effect of LED Light Spectra on Dill Cultivars’ Growth

**Figure 1:**
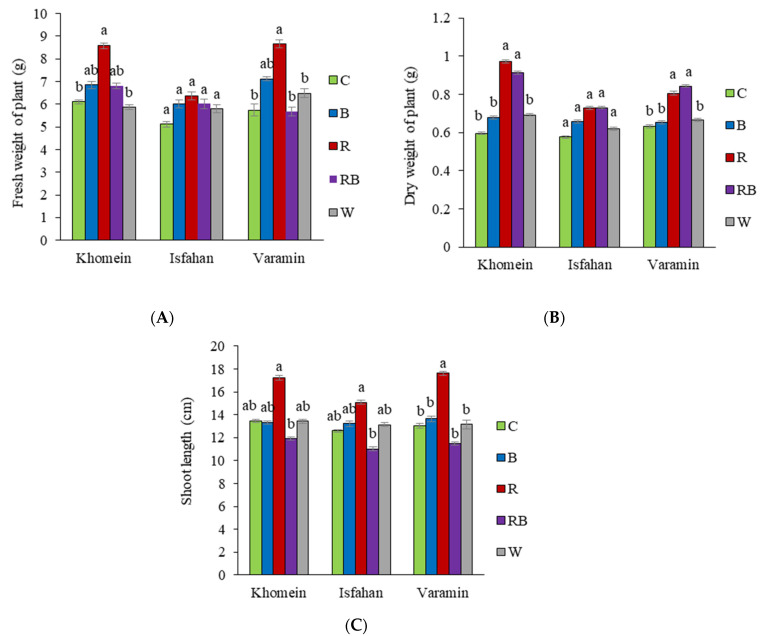
The interaction effect of different LED light treatments in each cultivar of dill on fresh weight of plant (**A**), dry weight of plant (**B**), and shoot length (**C**). The light treatments included greenhouse light as control (C), blue (B), red (R), red + blue (RB), and white (W). Significant differences between treatments are indicated by different letters (*p* < 0.05). Error bars represent the standard error of the mean, indicating the uncertainty of the mean values. Degrees of freedom of error (df) = 20 and sample number (n) = 25.

When exposed to R light, the fresh weight of the plants increased significantly in cultivars of Khomein and Varamin compared to the control light, but there was no statistically significant difference in the fresh weight of the Isfahan plants across light spectra compared to the control light (Figure 1A). The plants’ dry weight increased significantly under R and RB lights in the Khomein and Varamin cultivars compared to C, B, and W light. Furthermore, statistically significant differences were not observed in plant dry weight between the different LED light spectra with control light in the Isfahan cultivar (Figure 1B). The shoot length in Khomein and Isfahan cultivars significantly increased under R light treatment compared to RB light, while in the Varmin cultivar, all combinations of light were characterized by a shorter stem, except for R light (Figure 1C).

Alongside water, light is a crucial factor for plant growth, and extensive research has shown that different lighting conditions significantly affect plant morphology and physiology. These effects can vary widely among different plant species [25,26]. R light is particularly important, recognized as one of the most effective light colors for promoting typical growth and development [26,27]. For instance, Saleem et al. [28] found that R light enhances growth and biomass in *Brassica napus* L., while B light may inhibit growth due to radiation stress. The present study builds on these findings, revealing that plant growth is influenced not just by light quality but also by cultivar. Notably, the Isfahan cultivar did not exhibit significant increases in fresh and dry weight across various light treatments compared to the control light. In contrast, the Khomein and Varamin cultivars showed increased fresh weight under R light compared to the control light, indicating that R light effectively enhances biomass accumulation in these cultivars. The role of R light in promoting leaf structure development and photosynthetic machinery formation is well-documented [29]. Our results support the literature, emphasizing that while some cultivars may respond differently to light treatments, R light consistently promotes growth and biomass in certain cultivars. This variability highlights the importance of considering both light quality and plant cultivar for optimizing growth conditions.

Moreover, the influence of R light extends to stimulating the production of hormones that enhance stem internode length. Research indicates that uninterrupted R light can activate phytochromes, which regulate node length [30]. However, when combined with B light, R light can inhibit longitudinal growth [31]. Frąszczak et al. [32] reported that the tallest hypocotyls of dill were achieved with an R/B ratio of 70:10, suggesting that the limited presence of B light in this combination may mitigate its inhibitory effects on shoot growth [33]. Our findings corroborate this, as the Varamin cultivar exhibited increased shoot length under R light compared to other light spectra, further illustrating the nuanced interplay between light quality and plant growth responses.

### 2.2. Effect of LED Light Spectra on the Dill Cultivars Color Index

**Figure 2:**
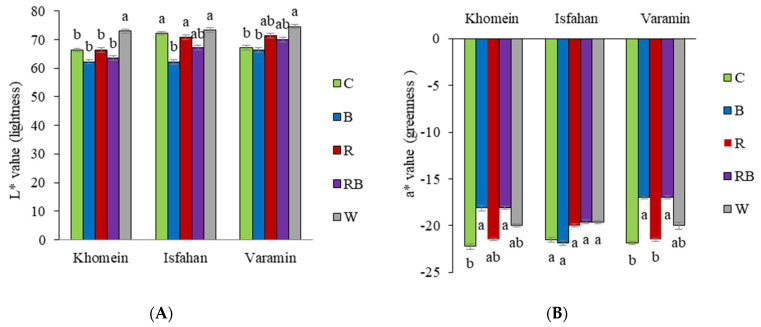

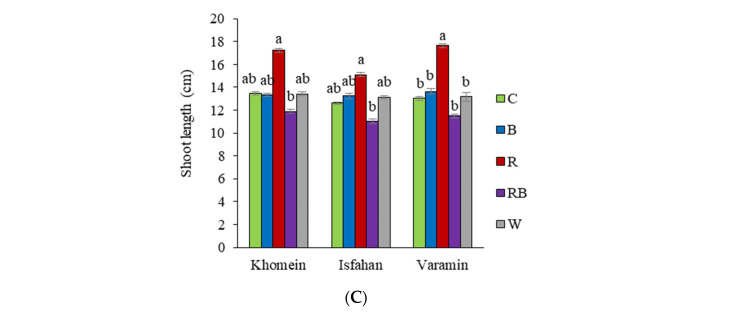
The interaction effect of different LED light treatments in each cultivar of dill on L* (**A**), a* (**B**), and b* (**C**) color indexes. The light treatments included greenhouse light as control (C), blue (B), red (R), red + blue (RB), and white (W). Significant differences between treatments are indicated by different letters (*p* < 0.05). Error bars represent the standard error of the mean, indicating the uncertainty of the mean values. Degrees of freedom of error (df) = 20 and sample number (n) = 25.

In the Isfahan cultivar, the L* index was higher under C, R, and W light compared to B light. In the Khomein cultivar, the L* index increased under W light compared to the C light. In the Khomein cultivar, the highest L* index was observed under W light compared to the other light spectra (C, B, R, RB) (Figure 2A). The a* index was significantly increased in the Khomein and Varamin cultivars under B and RB lights compared to the C light. However, there was no statistically significant change in the a* index in the Isfahan cultivar across the light spectra compared to the control light (Figure 2B). The b* index decreased significantly with B and RB light in all three cultivars, with the C and R light showing the highest values (Figure 2C).

An uptick in L* (lightness) shows that the leaf is turning more yellow and less green in color [34]. Owen and Lopez [35] discovered that in all tested cultivars of French lettuce, using an equal proportion of light in the colors R and B, as well as exclusively in B, reduced the L* index. Lee et al. [36] found that in the *Orostachys japonica* plant, the highest L* index was associated with green and W light, whereas the lowest index was associated with violet light. The current study also found that in all three cultivars, W light, which contains a green component in its spectrum, had the greatest L* index compared to the other light spectra. In both R and B lighting conditions, the Khomin cultivar had the lowest L* index, followed by the Isfahan and Varamin cultivars under B light.

The a* color coordinate is linked to the green hue of leaves [37], with the a* value indicating a decline in the original green color [Shoko]. Lee et al. [36] discovered that the a* index was highest when exposed to B light, followed by purple light, with the lowest index observed in green (G) light and the C group. In this study, the Khomein and Varamin cultivars showed the highest a* index under B and violet light conditions.

The b* color coordinate value, indicating blue + yellow opponents, shows that B light has negative values, while yellow has positive values [37]. The b* value tended to increment over capacity time, independent of light conditions, with the most noteworthy b* value seen in cultivars kept under W light for 9 days showing more articulated yellowing [34]. Tea leaves (cv. Hangjinya) exposed to a higher B light ratio exhibited a higher b* value (more yellow), possibly due to reduced levels of chlorophyll a, chlorophyll b, and the sum of chlorophyll a and chlorophyll b [38]. Similarly, the current study’s findings indicate an increase in the a* index in Khomein and Varamin cultivars under C, R, and W light, aligning with the results presented by Tian et al. [38].

### 2.3. Effect of LED Light Spectra on Photosynthesis Pigments in Dill Cultivars

**Figure 3:**
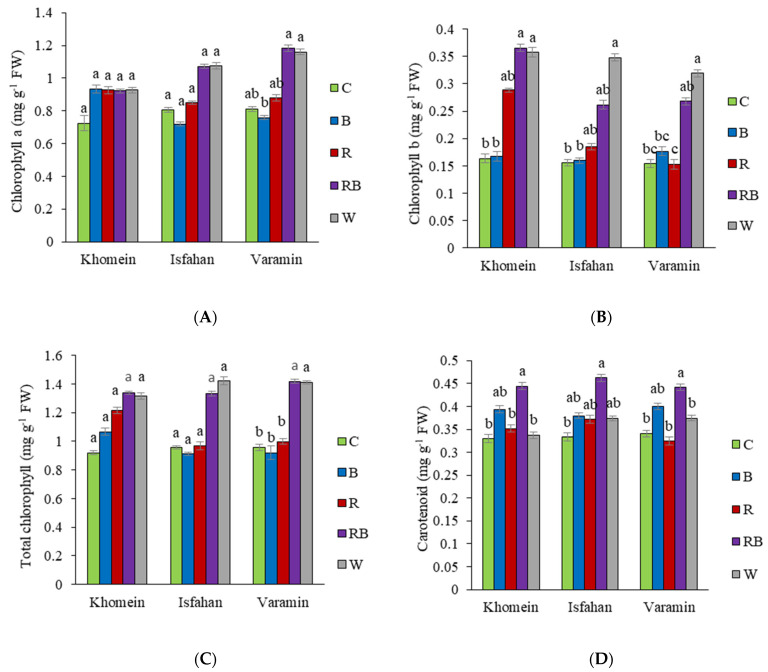
The interaction effect of different LED light treatments in each cultivar of dill on chlorophyll a (**A**), chlorophyll b (**B**), total chlorophyll (**C**), and carotenoid (**D**) concentrations. The light treatments included greenhouse light as control (C), blue (B), red (R), red + blue (RB), and white (W). Significant differences between treatments are indicated by different letters (*p* < 0.05). Error bars represent the standard error of the mean, indicating the uncertainty of the mean values. Degrees of freedom of error (df) = 20 and sample number (n) = 25.

The Varamin cultivar showed a significant increase in chlorophyll levels under RB and W light compared to B light, which was not significantly different from the control light. In contrast, the Khomein and Isfahan cultivars showed no statistically significant change in chlorophyll across the different LED lights (Figure 3A). When exposed to RB and W light, chlorophyll b levels increased significantly in cultivar Khomein compared to C and B light, while in cultivars Isfahan and Varamin, chlorophyll b increased under W light compared to the control light (Figure 3B). The Varamin cultivar’s total chlorophyll increased under RB and W light. However, the Khomein and Isfahan cultivars demonstrated no significant variation in total chlorophyll under different LED lights compared to the control light (Figure 3C). The carotenoid concentration in the Khomein cultivar increased under RB light compared to C, R, and W light, while other light types did not show significant differences from the control treatment. In the Isfahan cultivar, carotenoid concentration increased under RB light compared to the control treatment. For the Varamin cultivar, the highest carotenoid concentration was observed under RB light, while the lowest was noted under the C, R, and W light spectra compared to RB light (Figure 3D).

Hernandez et al. [39] found that varying proportions of a mix of R and B light, along with W light, significantly increased chlorophyll levels in tomatoes compared to pure R or B light. Our findings indicate that photosynthetic pigments are influenced not only by light spectrum but also by cultivar. In the Khomein and Isfahan cultivars, chlorophyll a and total chlorophyll levels did not show significant increases under the light treatments, whereas the Varamin cultivar exhibited higher chlorophyll content under RB and W light compared to B light. According to [20], the interaction between R and B light activates photoreceptors in plants, enhancing overall plant activity. Ahmadi et al. [40] reported that W light, followed by a combination of R and B light, led to substantial increases in chlorophyll a and total chlorophyll in lemons compared to pure R and B light. In the present study, the Khomein cultivar showed higher chlorophyll b levels under RB and W light compared to the control and B light. Additionally, Goswami and Mitra [4] demonstrated that RB light improved the expression of key photosynthesis-related genes AgpsbDII and AgRUBISCO in dill plants relative to pure B light. The utilization of different light spectra has also been shown to enhance carotenoid production. In our research, carotenoid levels varied among cultivars; specifically, the Khomein cultivar exhibited increased carotenoids under RB light compared to other light spectra. In the Isfahan cultivar, carotenoid levels increased under RB light compared to the control, with no significant differences observed among RB, B, R, and W light. This aligns with findings from Amoozgar et al. [41], who reported significantly higher carotenoid levels in lettuce grown under RB light.

### 2.4. Effect of LED Light Spectra on Physiological Parameters of Dill Cultivars

**Figure 4:**
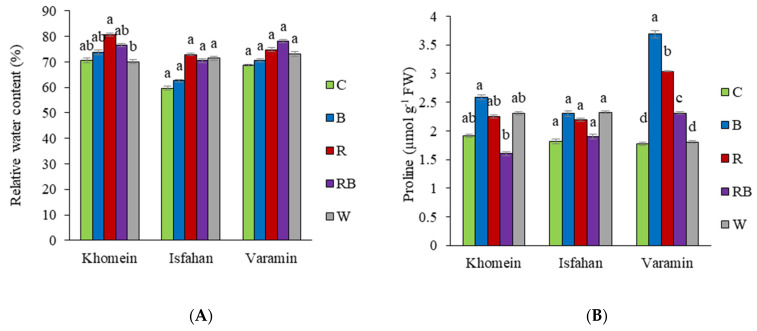

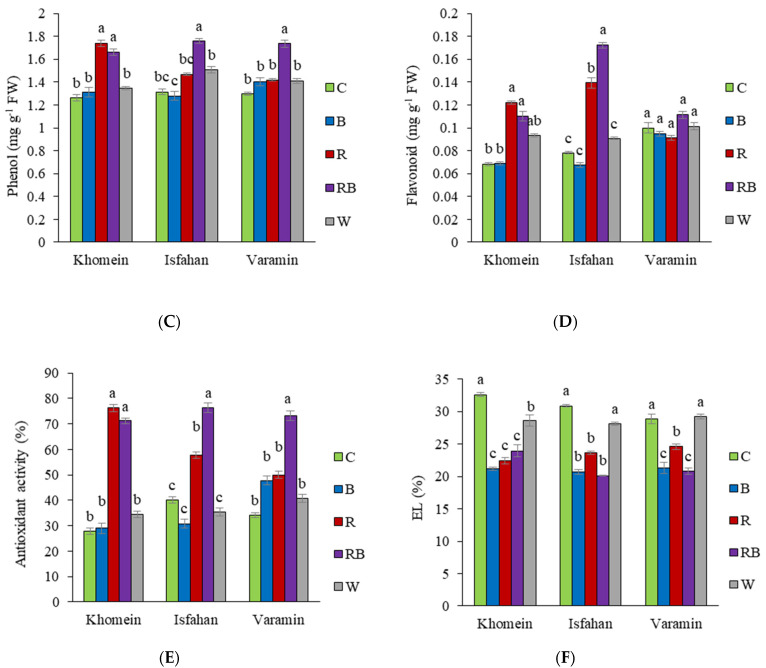
The interaction effect of different LED light treatments in each cultivar of dill on relative water content (**A**), proline (**B**), phenol (**C**), flavonoid (**D**), antioxidant activity (**E**), and EL (**F**). The light treatments included greenhouse light as control (C), blue (B), red (R), red + blue (RB), and white (W). Significant differences between treatments are indicated by different letters (*p* < 0.05). Error bars represent the standard error of the mean, indicating the uncertainty of the mean values. Degrees of freedom of error (df) = 20 and sample number (n) = 25.

In the Khomein cultivar, RWC showed a significant increase under R light compared to W light, while there was no significant difference compared to C, B, and RB light. No statistically significant change in RWC was observed in the Isfahan and Varamin cultivars across the light spectrum (Figure 4A). The concentration of proline in the Khomein and Isfahan cultivars did not show significant differences compared to the control under various light spectrums. However, in the Varamin cultivar, proline levels increased under B light compared to the other light conditions (Figure 4B). The Isfahan and Varamin cultivars exhibited the highest phenol levels under RB light, while the Isfahan cultivar showed the lowest levels under C, B, and R light. Additionally, the Varamin cultivar did not show significant differences in phenol levels across the other light spectra. However, the Khomein cultivar produced the most phenol under R and RB light compared with C, B, and W light (Figure 4C). The Khomein and Isfahan cultivars had significantly higher flavonoid concentrations under R and RB light compared to the control light, but there was no statistically significant difference in flavonoid concentration in the Varamin cultivar in various light spectra compared to the control light (Figure 4D). Antioxidant activity in the Khomein and Varamin cultivars was higher under R and RB light compared to other light spectra. In particular, the Varamin cultivar exhibited significantly greater antioxidant activity under RB light than under the other light spectra, while no significant differences were observed among the other light spectra (Figure 4E). EL increased significantly in all three cultivars under C and W light, with the minimum recorded for B, R, and RB light (Figure 4F).

Sabzalian et al. [20] found that RB light significantly improved the relative water content (RWC) in mint and basil leaves. In [42], it was noted that RB light treatment led to increased leaf thickness and a higher percentage of intercellular spaces, which likely enhances water movement within the leaves, contributing to improved RWC. The increased resilience of the mesophyll’s airspace on water movement is thought to facilitate this enhancement, possibly due to the expansion of intercellular spaces [43,44,45]. In contrast, the present study found no significant improvement in RWC for the Isfahan and Khomein cultivars compared to the control group. This discrepancy might be attributed to several factors. One possibility is that the environmental conditions and experimental setups, such as light intensity, duration, and the specific growth stages of the plants, could have influenced the outcomes. It is also possible that the light spectra used in the current study did not provide the same beneficial effects on RWC as those observed in mint and basil, indicating a potential species-specific response to light treatments.

Proline accumulation is a well-documented physiological response in plants under various stress conditions, such as high salinity, drought, and elevated temperatures. This amino acid plays a crucial role in enhancing plant tolerance and adaptation to these stressful environments [46,47]. Previous studies have demonstrated that proline levels increase under specific light conditions such as B light, particularly in tomato plants, when compared to R and G light [48]. In the current study, varying responses to different light spectra among the plant cultivars tested were observed. Specifically, the Khomein and Isfahan cultivars did not show significant differences in proline levels compared to the control, while the Varamin cultivar exhibited a notable increase in proline under B light. This discrepancy may be attributed to the inherent genetic and physiological differences among the cultivars. The Khomein and Isfahan cultivars might possess a more efficient metabolic pathway that minimizes proline accumulation under the tested light conditions, suggesting a robust mechanism for stress tolerance that does not rely heavily on proline buildup.

Lobiuc et al. [49] noted that the composition of phenolic acids in basil is primarily influenced by the ratio of R and B light, with the optimal ratio being 0.5:1. Additionally, in *Canavalia ensiformis*, a combination of R and B light at a 3:1 ratio was found to produce a higher total phenolic content compared to pure R, B, or W light [50]. Our research corroborates these findings, demonstrating that the total phenolic content in two cultivars, Isfahan and Varamin, was significantly higher under RB light compared to other light spectra. Notably, in the Khomein cultivar, both RB and R light treatments resulted in increased total phenolic content relative to other light conditions. This suggests that specific light combinations can effectively enhance phenolic compounds, which is crucial for plant defense mechanisms and overall health [51].

Light is an essential abiotic element that influences the accumulation of flavonoids and gene expression in different types of plant species [52]. Previous research on marjoram and oregano discovered that combined R and B light increased flavonoid levels in these plants [53]. Adil et al. [54] discovered that in *Cnidium officinale*, combining R and B light increased phenol and flavonoid secondary metabolites compared with other treatments. In keeping with the preceding findings, the current investigation found that flavonoids increased in Khomein and Isfahan cultivars under RB light compared to the control light.

Because light quality affects plant nutrient biosynthesis, modifying the spectrum of light can boost a plant’s antioxidant activities, resulting in more natural antioxidants in the daily diet [55]. Son and Oh [56] discovered that red leaf lettuce has better antioxidant activity when grown under both R and B LED lights (47% B light + 53% R light) compared to 100% R LED light. A comparable study on the dill plant found that the DPPH assays under RB light [4]. Our findings revealed that in all three cultivars, maximum antioxidant activity was observed under BR light.

Plants can suffer damage from prolonged exposure to continuous lighting [57]. Ginzburg and Klein [58] found that the EL in lettuce grown under 100% B and R light was significantly higher than in those grown under W light. Interestingly, a combination of R and B light resulted in reduced membrane leakage compared to single-color treatments. In contrast, the current study reveals that both control and W light produced the highest EL levels across all three dill cultivars, while B, R, and combined RB light treatments resulted in the lowest EL. This difference in findings may be because dill cultivars might be more sensitive to the stress induced by the control and W light spectra, leading to increased membrane instability and higher EL. Additionally, the specific light intensity and duration used in our experiment could have played a critical role in influencing the plants’ responses, suggesting that dill may require a different light regimen to minimize stress and maintain membrane integrity.

### 2.5. Effect of LED Light Spectra on Essential Oil Composition in Dill Cultivars

Analysis of samples using a GC-Mass device revealed 30 compounds in dill essential oil (Table 1). Apiole was observed in all treatments, followed by α-phellandrene, carvone, β-pinene, 3-carene, citronellol, citronellyl formate, and germacrene D. The amount of apiole increased the most in all three cultivars under R light. α-phellandrene levels increased in the Isfahan and Varamin cultivars under RB and B light. Compounds like α-gurjunene, aromadendrene, and camphor were unique to the Khomein cultivar, whereas thymol and myristicin were unique to the Isfahan cultivar (Table 1).

In this study, the most important and predominant chemical compound present in all treatments was apiole, which ranged from 24.41% (Khomein cultivar under B light) to 82.94% (Veramin cultivar under R light). The Varamin cultivar included the most apiole, whereas the Khomein cultivar contained the least. However, in all cultivars, R light dramatically enhanced apiole levels, whereas B light decreased them. Furthermore, prior research has shown that colored lights in varying quantities impact the function of the OMT1 gene (the gene responsible for apiole production) and produce apiole and dillapiole buildup via myristicin bifurcation metabolism [59].

In the Isfahan cultivar, α-phellandrenein levels were the highest (45.55%) under B light and lowest (2.66%) under W light. The Isfahan and Varamin cultivars exhibited α-phellandrenein in all light treatments. R light decreased, but RB light increased the amount of this molecule. Ahmadi et al. [40] found that applying W light to lemon balm reduced α-phellandrenein levels. In a similar study, R light decreased this chemical in dill plants, but RB light increased it [4].

The RB light increased dill ether in all cultivars in comparison to the C light, with the Varamin cultivar having the largest amount (14%). This compound was elevated in the Isfahan cultivar by 4.87% under W light and by 8.68% under B light. Previous research found that dill ether levels increased in dill plants under RB and W light but only slightly under pure B and R light [4]. Kim et al. [48] found that the unique aroma of dill stems from a synergistic link between α-phellandrene, dill ether, apiole, and myristicin.

Carvone cannot dissolve in water, yet it can dissolve in organic solvents. This compound is extremely valuable in the flavoring, aromatherapy, and air freshening industries, and it also exhibits antifungal, antioxidant, and insecticidal action, as well as anticancer characteristics [60]. Under violet light, the Varamin cultivar produced the most carvone (7.69%), but under the C light, it produced the least (0.03%). Furthermore, plants exposed to B light did not contain this component. Nguyen and Saleh [61] studied the essential oil of the mentha spicata plant under B, R, G, and C light and discovered that B light causes the elimination of this component in the plant.

All light spectra resulted in the formation of thymol in the Isfahan cultivar; however, this compound was not found in other cultivars. The largest quantity of thymol in the Isfahan cultivar was associated with W light exposure (12.96%). In a study on *Melissa officinalis*, Ahmadi et al. [40] discovered that the Isfahan and Ilam cultivars expressed higher levels of thymol due to W light therapy.

α-pinene was found in the Isfahan and Khomein cultivars under RB light. In a study on thyme cultivars, Tohidi et al. [17] discovered that this chemical was found in all of the examined cultivars when treated with R and B light.

Although all cultivars exposed to the C light included 3-carene, the maximum concentration was found in the Khomein cultivar (11.43%), and the use of other light spectra resulted in a reduction in or lack of this compound. In research conducted on two different types of balm mint plants in Ilam and Isfahan, the results were consistent with this experiment, with all light treatments causing a decrease in the amount of 3-carene compound in comparison to the C, whereas B and W light increased it in the Isfahan cultivar.

### 2.6. Heat Map and PCA of LED Light Spectra Impact on Dill Cultivars Growth and Physiological Characteristics

**Figure 5:**
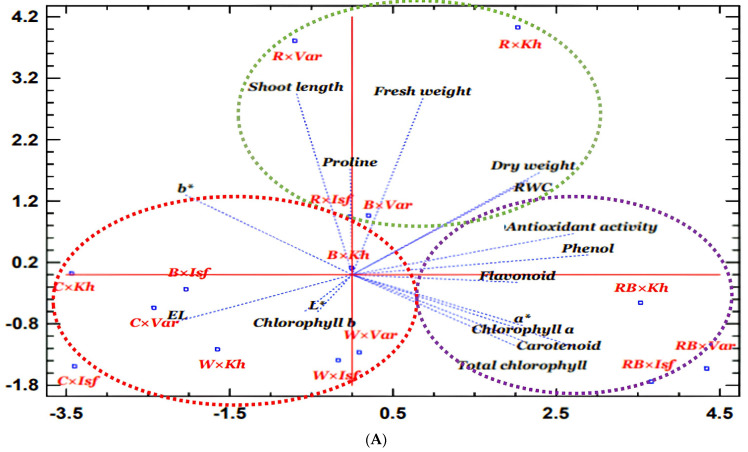

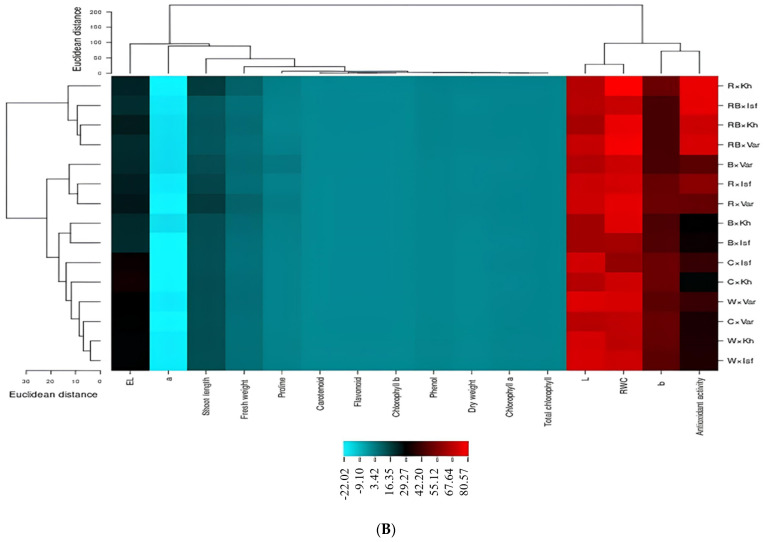
PCA (**A**) and heat map graph (**B**) of the interaction effect of different LED light treatments in each cultivar of dill on the growth and physiological characteristics of dill. The circles are colored purple for group A, green for group B, and red for group C in the PCA graph. Component 1 and component 2 in the PCA are 39% and 22%, respectively. Control light × Khomein cultivar (C × Kh), blue light × Khomein cultivar (B × Kh), red light× Khomein cultivar (R × Kh), red + blue light × Khomein cultivar (RB × Kh), white light× Khomein cultivar (W × Kh), control light × Isfahan cultivar (C × Isf), blue light × Isfahan cultivar (B × Isf), red light × Isfahan cultivar (R × Isf), red + blue light × Isfahan cultivar (RB × Isf), white light × Isfahan cultivar (W × Isf), control light × Varamin cultivar (C × Var), blue light×Varamin cultivar (BvVar), red light × Varamin cultivar (R × Var), red + blue light × Varamin cultivar (RB × Var), white light × Varamin cultivar (W × Var).

The biplot graph illustrates that the impact of light treatments on characteristics can be classified into three groups. The A group circle in purple, which includes RB light on all three cultivars, increased biochemical and physiological traits (antioxidant activity, phenol, flavonoid, carotenoid, chlorophyll a, and total chlorophyll). The B group circle in green, which includes R light on all three cultivars and the Varamin cultivar in B light, increased morphological and physiological traits (fresh and dry weight of the plant, shoot length, RWC, and proline), whereas the C group circle in red, which includes W light and C light treatment in all three cultivars and B light in the Isfahan and Khomein cultivars, increased some physiological traits (chlorophyll b, EL and L*, a*, b* index) (Figure 5A).

The heat map graph shows that among the traits studied, RWC, antioxidant activity, L* and b* index, EL, shoot length, and plant fresh weight were most affected by the treatments. The treatments had the smallest effect on the a* index, carotenoid, flavonoid, and chlorophyll b. The Isfahan and Varamin cultivars exhibited the greatest antioxidant potential when exposed to RB light, followed by the Khomein cultivars under R light. Furthermore, the lowest RWC was found in all three cultivars under the C light. All three cultivars showed an increase in the b* index under R light and C conditions. The antioxidant activity and the b* index were placed in separate clusters. The RWC and L* indices were also separated into their own clusters. RWC values were highest in the Khomein and Varamin cultivars under R and RB light and lowest in the Isfahan cultivar under C, B, and RB light. Under W light, all three cultivars exhibited the highest L* index values. Plant fresh weight and shoot length were separated and increased in the Khomein and Varamin cultivars under R light (Figure 5B).

### 2.7. Heat Map and PCA of LED Light Spectra Impact on Dill Essential Oil in Different Cultivars

**Figure 6:**
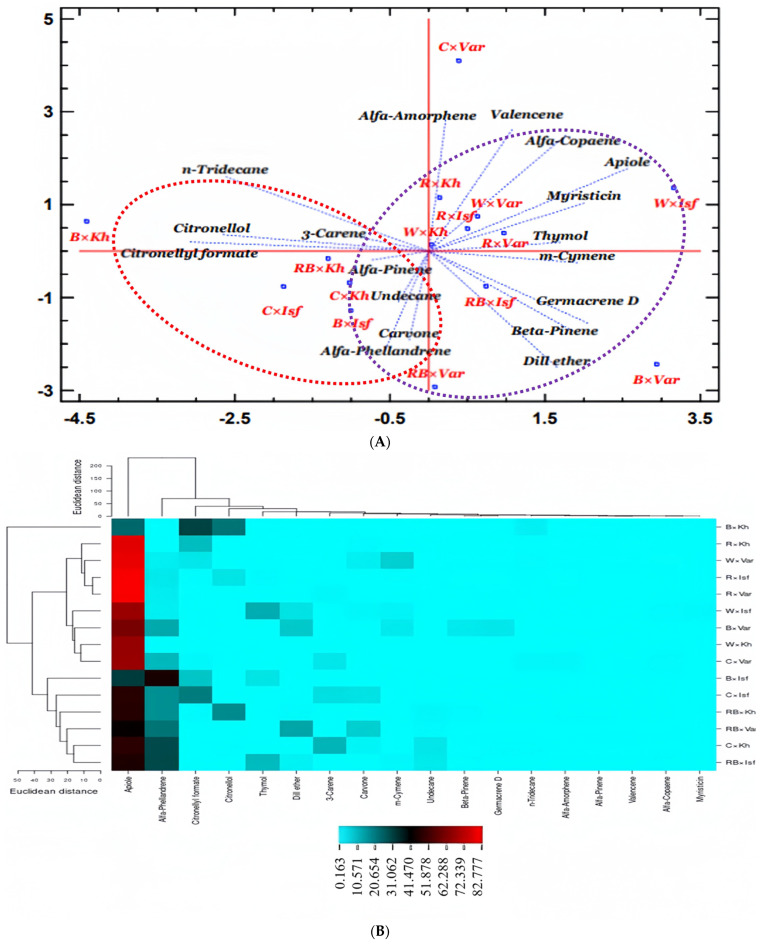
PCA (**A**) and heat map graph (**B**) of the interaction effect of different LED light treatments in each cultivar of dill on the essential oil. Component 1 and component 2 in the PCA are 21% and 18%, respectively. The circles are colored purple for group A, and red for group B in the PCA graph. Control light × Khomein cultivar (C × Kh), blue light × Khomein cultivar (B × Kh), red light × Khomein cultivar (R × Kh), red + blue light × Khomein cultivar (RB × Kh), white light × Khomein cultivar (W × Kh), control light × Isfahan cultivar (C × Isf), blue light × Isfahan cultivar (B × Isf), red light × Isfahan cultivar (R × Isf), red + blue light × Isfahan cultivar (RB × Isf), white light × Isfahan cultivar (W × Isf), control light × Varamin cultivar (C × Var), blue light × Varamin cultivar (B × Var), red light × Varamin cultivar (R × Var), red + blue light × Varamin cultivar (RB × Var), white light × Varamin cultivar (W × Var).

According to the PCA diagram, the exposure of the Isfahan cultivar to RB light increased the levels of α-copaene, apiole, thymol, m-cymene, β-pinene, dill ether, α-pinene, undecane, carvone, and α-pinene, as indicated by the purple circle (A group). Under RB light in the Khomein cultivar, α-pinene, undecane, carvone, α-phellandrene, citronellol, and citronellyl increased, as shown by the red circle (B group) (Figure 6A).

According to the heatmap graph, apiole essential oil was more affected by the treatments and was assigned to a separate cluster. Under R light, the Isfahan and Varamin cultivars had the most apiole (red), whereas, under RB light, they had the least. α-phellandrene was increased in the Isfahan cultivar using B and RB light. Citronellyl formate and citronellol were found in the highest concentrations in the Khomein cultivar under R light. The treatments had a lower effect on the essential oil compounds α-pinene, valencene, α-copaene, and myristicin (Figure 6B).

## 3. Materials and Methods

### 3.1. Design of Experiments, Cultivation, and Treatment Application

The research was carried out at Isfahan University of Technology’s research greenhouse, where the average daily temperature was around 25 ± 2 °C and the average night-time temperature was 17 ± 2 °C. The aim was to make a comparison of the impact of various spectra produced by LED lights on the secondary metabolic composition and the morphological and physiological parameters of three dill cultivars. The factorial experiment used a complete randomized design (CRD) with three replications. LED light treatments at five levels (greenhouse light as C, B, R, RB, and W) were applied to Khomein, Isfahan, and Varamin dill cultivars. The geographical location specifications of the cultivars of dill are presented in Table 2. Seeds of the dill cultivar (*Anethum graveolens*) were acquired from the Pakan Bazr firm in Isfahan, according to the information in Table 3. The seeds were planted in cultivation boxes with a depth of 5 cm and lengths and widths of 60 and 30 cm, respectively. Each growing box had an area of half a meter and contained 25 plants with 3 replications for each treatment. The cultivation bed included a blend of farm soil and sand in a proportion of (V/V 1:2). In the greenhouse, the dill seeds were grown in accordance with prescribed conditions. Treatments included natural light (C), 100% B light, 100% R light, 25% R + 75% B light, and 100% W light with wavelengths of R light λ = 650–665 nm, B light λ = 460–475 nm, and W light λ = 380–760 nm. LED panels were set 50 cm from the plant surface, and seedlings were exposed to light for 20 h every day from 7 AM to 3 AM, with an overall photosynthetic photon flux density (PPFD) of 100 μmol m^−2^s^−1^. This PPFD is for W, R, B, and RB light. The natural light period was from 6 AM to 6 PM with PPFD 260 μmol m^−2^s^−1^. During the experiment, the weeds were thinned and removed manually. To enhance the plants throughout the growth stage, a complete fertilizer with 0.5 g L^−1^ concentration was administered as irrigation fertilizer. Irrigation was performed as required. The following parameters were assessed 45 days after the initiation of light treatment when the plants reached a height of 10 cm.

Generally, biometric and chemical analyses were performed on all observations of 25 plants, with each measurement taken in triplicate.

### 3.2. Measured Parameters

#### 3.2.1. Plant Growth Parameters

The plants were carefully collected by hand, ensuring that the roots were gently pulled from the soil to minimize damage. After harvesting, the plants were thoroughly washed to remove any soil and debris. This method ensures that the fresh weight measurements are accurate and reliable. The fresh weight of the plants, including shoot and root, was quantified using a precise scale that is sensitive to 0.01 g. After undergoing a two-day drying process, they were maintained at a stable weight. After being heated to a temperature of 70 °C, the initial weight and the weight after drying of the plants were determined. Shoot length was measured in centimeters using a ruler.

#### 3.2.2. Color Index

The lightness or darkness (L*), the red or greenness (a*), and the yellow or blueness (b*) color index of dill were measured in pixels. A Canon EOS 6D digital camera was used to capture photographs of the leaves, ensuring that they were in a consistent posture and under uniform lighting conditions. The Photoshop CS5 application was used to apply image processing and coding techniques to extract color components and other relevant data from the photographs [62].

#### 3.2.3. Total Carotenoid and Chlorophyll Content of the Leaves

The 0.2 g leaf samples were mixed together in acetone that was 80% pure. The extract was centrifuged at a speed of 12,000 revolutions per min at ambient temperature for 5 min. The supernatant’s optical density was determined using a UV 160A spectrophotometer made by Shimadzu Corp in Kyoto, Japan, at 663 nm, 646 nm, and 470 nm. Chlorophyll and carotenoid levels were determined using the formula from Lichtenthaler et al. [63]; formula in mg g^−1^ FW.

#### 3.2.4. The Leaf Relative Water Content

The leaves’ fresh weight (FW) was promptly measured. Afterward, filtered water was used to soak the leaves for 12 h at a moderate room temperature. Leaves containing too much moisture were dried using a towel to remove surface water, and the weight obtained was recorded as the turgid weight (TW). The wet leaves were dehydrated in an oven at 60 °C to find the dry weight (DW), and the relative water content (RWC) was computed using López-Serrano et al.’s [64] formula.

$$RWC=\frac{\left(FW-DW\right)}{\left(TW-DW\right)}\times 100$$

#### 3.2.5. Concentrations of Proline

Bates et al. [65] explained the ninhydrin assay for measuring proline levels. Leaf samples were mixed at 4 °C with a 3% sulfosalicylic acid solution for homogenization. Next, the solution was placed in a centrifuge and spun at 5000 revolutions per min for 20 min. The liquid was mixed with a solution that included 2.5% ninhydrin, 60% phosphoric acid (volume/volume), and 1 mL of glacial acetic acid (100% concentration). Absorbance was measured through the use of a wavelength of 518 nm. Proline concentrations were expressed in micromoles per gram of fresh weight (µmol g^−1^ FW).

#### 3.2.6. Total Phenolics

A solution of 1 mL extract (prepared by homogenizing 1 g of fresh leaf tissue in 80% methanol) at 100–500 µg mL^−1^, 2.5 mL of a solution containing 10% Folin-Ciocalteu reagent was introduced into the blend. Following a 5-min delay, a solution of 2.0 mL Na_2_CO_3_ (7.5% concentration; 1 mL) and 1 mL distilled water was incorporated into the mixture. The resultant mixture was kept at 50 °C for 10 min during incubation. The specimen was then cooled, and measurement of the absorbance was conducted with a UV Spectrophotometer at a 730 nm wavelength, with a blank sample devoid of extract acting as a standard. The measurements were presented in mg gallic acid g^−1^ of fresh extract [66].

#### 3.2.7. Flavonoid Concentrations

The flavonoid content was assessed using the methodology described by Yazdizadeh Shotorbani [67], with minor changes. A solution was prepared by combining 0.2 mL of leaf extract (prepared by homogenizing 1 g of fresh leaf tissue in 80% methanol) with 0.8 mL of distilled water. To this mixture, 1 mL of a methanol solution containing 2% AlCl_3_ and 5% acetic acid was added. After letting the solution sit, the determination of absorption at 430 nm was taken at room temperature for a duration of 10 min, using the reactant as the sample blank. A quercetin standard sample was used to create a calibration curve. The amount of flavonoids in the samples was measured in mg of quercetin equivalents for every g of fresh leaf weight.

#### 3.2.8. Antioxidant Activity

Following the procedure set out by Koleva et al. [68], the antioxidant activity of dill leaves was evaluated. First, a test tube containing 200 μL of the provided solution along with 800 μL of Tris-HCl buffer at a pH of 7.4 was set up. Following this, a brief mixing was conducted after adding 1 mL of DPPH solution. Afterward, the blend was left in dimness at normal room temperature. After 30 min, a spectrophotometer was employed to determine the amount of light absorbed by the DPPH solution at 517 nm wavelength. A reference was made using a solution containing Tris-HCl buffer and ethanol. The absorbance recorded while injecting the analytical sample was labeled as As, while the measured absorbance with ethanol instead was known as Ac. The calculation for percentage of inhibition was carried out using the equation below:

$$Inhibition\;ratio\;(\%)=(\frac{\left(Ac-As\right)}{Ac})\times 100$$

#### 3.2.9. Determination of Electrolyte Leakage (EL)

After three complete cleanings, the leaf segments (0.2 g) were added to 6 mL of purified water. During the initial stage of the rehydration process, electrical conductivity (Eci) was assessed for the first time. Subsequently, the segment-containing tubes were transferred to a 25 °C, dark environment. Afterward, measurements (Ecf) were taken at intervals of 0.5, 1.5, 3.5, 7.5, and 22.5 h throughout the rehydration process. Following the aforementioned measurements, the samples were autoclaved and refrigerated to 25 °C. The sample’s overall electrical conductivity (Ect) was then calculated using Bajji et al.’s [69] formula.

$$EL\;(\%)=\frac{\left(Ecf-Eci\right)}{\left(Ect-Eci\right)}\times 100$$

#### 3.2.10. Percentage and Components of Essential Oils

The steam distillation process and a Clevenger machine were used to extract the essential oil (MS-E104, USA). This gadget consists of three primary components: a glass balloon, an oil separation tube, and a coolant. The materials dried in the open air were crushed and put into a one-liter flask, which was then filled with approximately one liter of distilled water. The device was then switched on for 5 h until the extracted essential oil emerged as a yellow oily layer on the water’s surface and was collected in a tube [53]. After weighing the essential oil, the weight percentage was computed using the formula presented by Sarfaraz et al. [53].

#### 3.2.11. Essential Oil Analysis

The components of the essential oil were evaluated using gas chromatography-mass spectrometry (GC-MS) analysis. For the analysis, an experiment was performed with an Agilent 7890 gas chromatograph that had an HP-5 MS capillary column attached. The temperature settings of the oven, injector, and detector were carefully controlled during the process. The initial oven temperature was 60 °C for 4 min, and for the next steps, 260 °C was applied. The injector and detector temperatures were 290 °C and 300 °C, respectively. Helium was applied as the carrier gas with a flow rate of 2 mL/min. The instrument was connected to an Agilent 5975C mass spectrometer for specific detection. An ionization voltage of 70 eV and an ion source temperature of 200 °C were used for the mass spectrometry. To identify the essential oil components, retention indices were compared with retention indices (RI) of C5–C24 n-alkanes as well as available data in the literature, along with mass spectra comparisons with databases like NIST 08 and Wiley 275 [70].

### 3.3. Statistical Analysis

The investigation involved an experiment with a factorial design and a complete randomized design with three replications. SAS Ver 9.4 was used to analyze the data. The information was evaluated through two-way ANOVA, and the mean values were compared for significance using the LSD examination with a significance level of p less than 0.05. Statgraphics Centurion, Version XVI, was used to perform the principal component analysis (PCA).

## 4. Conclusions

In general, while all of the light spectra used improved the plants’ condition in most traits in comparison to the C, RB light outperformed the other light spectra in terms of photosynthesis pigments, phenol, flavonoid, and antioxidant capacity. Khomein and Varamin had increased weights, both fresh and dry, and RWC, than the Isfahan cultivar, which had higher flavonoids. Apiole essential oil was more impacted by the treatments. Under R light, the Isfahan and Varamin cultivars had the highest amount of apiole, whereas, under RB light, they had the least amount. α-phellandrene was found in the Isfahan cultivar under B and RB light. Citronellyl and citronellol were identified in the highest amounts in the Khomein cultivar under R light. As a result, the RB light and Khomein cultivars showed improved physiological traits, whereas RB and R light in the Varamin and Isfahan cultivars are recommended for higher essential oil compositions to produce functional food.

## Figures and Tables

**Table 1:** Mean essential oil compositions of dill cultivars under various LED light spectra.

Compound	RI^a^	Khomein					Isfahan					Varamin				
		C	B	R	RB	W	C	B	R	RB	W	C	B	R	RB	W
2-butyl-octanol	798	nd	nd	2.82	nd	nd	nd	nd	nd	nd	nd	nd	nd	nd	8.22	nd
α-pinene	939	nd	nd	nd	0.49	0.66	nd	nd	nd	0.07	nd	nd	nd	nd	nd	nd
β-pinene	976	0.62	nd	nd	0.73	0.56	nd	nd	0.37	0.52	0.22	nd	3.48	nd	nd	nd
α-phellandrene	1004	28.97	nd	nd	17.85	17.7	17.6	45.55	3.62	29.53	2.66	10.85	13.59	3.04	22.19	2.79
3-carene	1018	11.43	nd	nd	nd	6.46	5.11	nd	0.16	nd	nd	4.04	nd	1.36	nd	nd
m-cymene	1024	nd	nd	nd	nd	nd	nd	nd	0.44	1.02	2.16	nd	3.45	nd	nd	7.67
γ-terpinene	1060	nd	nd	nd	0.34	nd	nd	nd	nd	0.64	nd	nd	nd	nd	nd	nd
terpinolene	1086	nd	nd	nd	6.15	nd	nd	nd	0.72	3.03	nd	nd	nd	nd	nd	nd
undecane	1100	3.98	nd	nd	1.38	nd	nd	nd	nd	3.44	nd	nd	nd	nd	0.85	nd
n-tridecane	1300	0.6	2.82	nd	nd	nd	0.63	nd	nd	nd	nd	1.43	nd	nd	nd	0.7
dill ether	1118	nd	nd	nd	0.78	nd	nd	nd	nd	2.58	4.87	nd	8.68	nd	14	nd
camphor	1126	nd	nd	3.64	0.7	nd	nd	nd	nd	nd	nd	nd	nd	nd	nd	nd
β-citronellol	1196	nd	nd	nd	nd	nd	nd	nd	nd	nd	1.45	nd	nd	0.13	nd	nd
citronellol	1225	0.48	22.23	nd	18.5	1.76	nd	nd	4.52	nd	nd	nd	nd	nd	nd	nd
carvone	1243	1.88	nd	0.67	0.53	2.94	4.77	nd	0.24	0.23	1.32	0.03	nd	0.16	7.69	2.78
citronellyl formate	1261	nd	30.45	10.27	0.91	nd	20.92	8.87	0.39	nd	nd	1.09	nd	nd	nd	3.99
thymol	1290	nd	nd	nd	nd	nd	nd	4.49	0.88	10.69	12.96	nd	nd	nd	nd	nd
β-cubebene	1348	0.35	nd	nd	0.26	nd	nd	nd	nd	0.28	nd	1.11	nd	0.24	nd	nd
α-copaene	1378	0.04	nd	nd	0.03	nd	nd	nd	nd	0.55	0.74	0.85	nd	nd	nd	0.34
β-elemene	1395	0.08	nd	nd	0.06	nd	nd	nd	nd	0.08	nd	nd	nd	nd	nd	nd
α-gurjunene	1407	0.02	nd	nd	nd	0.18	nd	nd	nd	nd	nd	nd	nd	nd	nd	nd
aromadendrene	1438	0.77	nd	nd	0.07	nd	nd	nd	nd	nd	nd	nd	nd	nd	nd	nd
germacrene D	1478	0.39	nd	nd	0.27	0.41	nd	nd	0.55	nd	0.43	nd	3.99	0.48	nd	0.12
valencene	1491	0.18	nd	0.46	0.14	nd	nd	nd	nd	nd	0.2	0.64	0.22	0.15	nd	nd
α-amorphene	1511	0.18	nd	nd	0.61	nd	nd	nd	0.64	0.14	0.14	1.61	nd	nd	nd	nd
δ-cadinene	1523	0.08	nd	nd	nd	nd	nd	nd	nd	0.33	nd	nd	nd	nd	nd	nd
myristicin	1523	nd	nd	nd	nd	nd	nd	nd	0.18	nd	1.04	nd	nd	nd	nd	nd
caryophyllene oxide	1584	0.09	nd	nd	0.69	nd	nd	nd	nd	nd	nd	0.56	nd	nd	nd	nd
apiole	1705	49	24.41	79.23	48.1	66.61	48.14	31.35	82.67	46.83	67.18	66.91	61.56	82.94	43	80.58
phytol	2100	nd	nd	nd	nd	nd	nd	nd	0.3	nd	0.57	1.35	1.98	0.68	nd	nd
others	_	0.45	2.3	2.4	1.41	2.73	2.83	nd	1.75	0.04	1.94	0.02	3.05	3.05	4.05	1.03
Essential oil content (%)		0.23	0.26	0.24	0.27	0.33	0.19	0.28	0.27	0.34	0.29	0.24	0.26	0.25	0.28	0.33

The information was arranged according to the retention index (RI) of components. Treatments included greenhouse light as control (C), blue (B), red (R), red + blue (RB), and white (W) and cultivars of dill, including Khomein, Isfahan, and Varamin. RI^a^: retention indices (RIs) as established using a homologous series of n-alkanes (C5–C24) on a DB-5 MS column. nd: not detected. Literature indexes were obtained from the NIST database (2009).

**Table 2:** Name and geographical location of the different dill cultivars.

Cultivar	Longitude	Latitude	Altitude (cm)
Khomein	50°5′ E	33°43′ N	1815
Isfahan	51°40′ E	32°40′ N	1575
Varamin	51°38′ E	35°19′ N	920

**Table 3:** Information on seeds.

Cultivars	Production Area	Height of the Area Above Sea Level	Germination (%)	Purity (%)
Khomein	Khomein	1800	75	90
Isfahan	North Braan	1510	80	90
Varamin	Varamin	1000	84	94

## Data Availability

The data presented in this study are available on request from the corresponding authors.
